# Targeted nutritional intervention attenuates experimental lung cancer cachexia

**DOI:** 10.1002/jcsm.13520

**Published:** 2024-07-04

**Authors:** Wouter R. P. H. van de Worp, Jan Theys, Cecile J. A. Wolfs, Frank Verhaegen, Annemie M. W. J. Schols, Ardy van Helvoort, Ramon C. J. Langen

**Affiliations:** ^1^ Department of Respiratory Medicine, NUTRIM – Institute of Nutrition and Translational Research in Metabolism Maastricht University Medical Center Maastricht The Netherlands; ^2^ Department of Precision Medicine, GROW – Institute for Oncology and Reproduction Maastricht University Medical Center Maastricht The Netherlands; ^3^ Department of radiation Oncology (Maastro), GROW – Institute for Oncology and Reproduction Maastricht University Medical Centre+ Maastricht The Netherlands; ^4^ Danone Nutricia Research Utrecht The Netherlands

**Keywords:** Cachexia, Intervention, Lung cancer, Muscle wasting, Nutrition

## Abstract

**Background:**

Cachexia, a syndrome with high prevalence in non‐small cell lung cancer patients, impairs quality of life and reduces tolerance and responsiveness to cancer therapy resulting in decreased survival. Optimal nutritional care is pivotal in the treatment of cachexia and a recommended cornerstone of multimodal therapy. Here, we investigated the therapeutic effect of an intervention diet consisting of a specific combination of high protein, leucine, fish oil, vitamin D, galacto‐oligosaccharides, and fructo‐oligosaccharides on the development and progression of cachexia in an orthotopic lung cancer mouse model.

**Methods:**

Eleven‐week‐old male 129S2/Sv mice were orthotopically implanted with 344P lung epithelial tumour cells or vehicle (control). Seven days post‐implantation tumour‐bearing (TB) mice were allocated to either intervention‐ or isocaloric control diet. Cachexia was defined as 5 days of consecutive body weight loss, after which mice were euthanized for tissue analyses.

**Results:**

TB mice developed cachexia accompanied by significant loss of skeletal muscle mass and epididymal fat mass compared with sham operated mice. The cachectic endpoint was significantly delayed (46.0 ± 15.2 vs. 34.7 ± 11.4 days), and the amount (−1.57 ± 0.62 vs. −2.13 ± 0.57 g) and progression (−0.26 ± 0.14 vs. −0.39 ± 0.11 g/day) of body weight loss were significantly reduced by the intervention compared with control diet. Moreover, systemic inflammation (pentraxin‐2 plasma levels) and alterations in molecular markers for proteolysis and protein synthesis, indicative of muscle atrophy signalling in TB‐mice, were suppressed in skeletal muscle by the intervention diet.

**Conclusions:**

Together, these data demonstrate the potential of this multinutrient intervention, targeting multiple components of cachexia, as integral part of lung cancer management.

## Introduction

Lung cancer is the second most commonly diagnosed cancer and accounts for 18% of total cancer deaths, which makes lung cancer the leading cause of cancer‐related deaths worldwide.^1^ Approximately 85% of the lung cancer patients suffer from non‐small cell lung carcinoma (NSCLC) and are predominantly diagnosed at an advanced stage.^2^ More than half of these patients with advanced stage NSCLC suffer from cancer cachexia,^3^ a complex multifactorial syndrome characterized by pronounced loss of skeletal muscle mass.^4^ Cancer cachexia has a negative impact on physical function, symptom burden and survival, negatively affecting quality of life.^5^ In addition, cancer cachexia contributes to treatment delays and treatment‐related side effects, limiting the efficacy of promising cancer therapies.^6^, ^7^ Effective intervention strategies targeting cancer cachexia are an unmet medical need, but their development is imperative to increase the efficacy of and systemic tolerance to cancer treatment and consequently improve patient prognosis.

Optimal nutritional care is pivotal in the treatment of cancer cachexia and is recommended as a cornerstone of multimodal therapy.^8^ Nutritional interventions extend beyond merely adjusting energy intake but can be tailored to target disease‐related characteristics, including impaired muscle protein turnover and chronic inflammation which accompany cancer cachexia and may drive muscle wasting.^9^

Extensive studies on protein metabolism in cancer patients are scarce, but post‐absorptive whole body protein turnover appears to be elevated in cancer patients compared with healthy individuals.^10^ Moreover, muscle protein turnover is disrupted, favouring protein breakdown in patients with cancer cachexia.^11^ Maintenance of muscle mass requires post‐prandial protein synthesis to exceed protein breakdown sufficiently to balance the net loss of protein in the post‐absorptive state. As nutritional intake is often reduced in cachectic cancer patients,^12^ it is expected that the provision of extra essential amino acids, in the form of orally ingested protein, would stimulate maintenance and accretion of muscle protein. Indeed, several studies showed that high protein diets can stimulate muscle protein synthesis in cancer patients.^13^, ^14^ Such effects have mainly been attributed to whey proteins.^15^ Due to its favourable amino acid profile and the fast absorptive kinetics, it is anticipated that whey protein constitutes a potent protein source to stimulate protein synthesis and thus limit muscle protein loss in cancer patients.

Among the essential amino acids, branched‐chain amino acids (BCAAs) and especially L‐leucine are unique for their critical role in muscle protein metabolism by stimulating protein synthesis and inhibiting protein degradation.^16^ While there is no evidence from long term intervention studies with L‐leucine in human cancer cachexia patients, in multiple experimental animal models of cancer cachexia, it has been demonstrated that the tumour‐induced muscle wasting was attenuated by L‐leucine supplementation.^9^ This beneficial effect of L‐leucine is mediated by the direct modulation of muscle protein synthesis and degradation, providing a mechanistic basis for considering L‐Leucine in a multitargeted, multinutrient intervention.

Vitamin D is increasingly recognized as a co‐factor that might play an important role in maintaining muscle function. Low vitamin D status is associated with an increased risk of muscle weakness.^17^ Moreover, vitamin D deficiency is very common in patients with cancer cachexia.^18^ Because preservation of vitamin D status can easily be accomplished through dietary supplementation, this warrants its inclusion as part of a multinutrient intervention.

Cancer cachexia is typically accompanied by chronic inflammation.^4^ Patients with cancer cachexia often present with elevated serum inflammatory markers such as C‐reactive protein, interleukin‐6, and interleukin‐8.^19^ Cancer‐induced systemic inflammation and the consequent host‐derived catabolic stimuli have been implicated in cachexia. Therefore, it is expected that nutritional supplementation with nutrients that exhibit anti‐inflammatory properties would maintain muscle protein turnover and immunological state. Fish oil, which is rich in omega‐3 long chain polyunsaturated fatty acids (n‐3 lcPUFA) eicosapentaenoic acid (EPA) and docosahexaenoic acid (DHA), may have anti‐catabolic affects by reducing systemic inflammation.^20^ Besides preclinical evidence, this notion is supported by clinical trials reporting clinically and statistically significant effects of fish oil/EPA on systemic inflammation, body weight, muscle mass, and/or performance status.^21^ Accordingly, these studies provide a rationale for considering fish oil/EPA as an effective component in the multinutrient therapy.

Immune modulating activities have been attributed to prebiotic non‐digestible oligosaccharides such as galacto‐oligosaccharides (GOS) and fructo‐oligosaccharides (FOS) based on observations in several clinical trials and studies using preclinical models.^22^, ^23^ These oligosaccharides may block or activate specific receptors on immune cells leading to improved immune responses. In addition, oligosaccharides are fermented by colonic bacteria into short‐chain fatty acids (SCFAs).^24^ SCFAs have been shown to exert anti‐inflammatory properties.^25^ In addition, in mice bearing C26 tumours, diet supplemented with GOS and FOS significantly reduced skeletal muscle mass loss.^26^ Accordingly, GOS and FOS constitute potential candidates for multinutrient interventions targeting cancer cachexia.

Based on single nutrient supplementation evidenced above, we hypothesized that their combined effects as part of a multitarget, multinutrient intervention is a potent strategy to treat cancer cachexia. Therefore, we investigated the therapeutic effect of an intervention diet consisting of a specific combination of high protein (whey), L‐leucine, vitamin D, fish oil, GOS, and FOS on the development of cachexia in an orthotopic lung cancer mouse model.^27^

## Methods

### Ethical approval

This study was conducted in accordance with institutional guidelines for the care and use of laboratory animals of the University of Maastricht, and all animal procedures related to the purpose of the research were approved by the local Animal Welfare Body under an Ethical licence provided by the national competent authority (Centrale Commissie Dierproeven, CCD), securing full compliance to the European Directive 2010/63/EU for the use of animals for scientific purposes.

### Animals

Fifty‐one male 129S2/Sv mice of 11 weeks (129S2/SvPasCrl, Charles River Laboratories, Germany) were socially housed (*n* = 3) in GM500 IVC cages (Tecniplast, Buguggiate, Italy) with Corn cob bedding (JRS Lignocel, Rosenberg, Germany), shelter and nesting material in a climate‐controlled room (12:12 dark–light cycle with a constant room temperature of 21 ± 1°C). Mice were given *ad libitum* access to food (AIN‐93 M, Bio Services BV, Uden, the Netherlands) and drinking water [autoclaved, softened and acidified (pH = 2.5)]. After 1 week of acclimatization, animals were randomly allocated to either sham operated group or tumour‐bearing (TB) groups (detailed information provided in flowchart, *Figure* S1). Seven days post‐tumour inoculation, tumours were scored for location and size using the Micro Cone Beam Computed Tomography (μCBCT). If the primary tumour did not appear in the lung, mice were excluded. TB mice were allocated to either control diet (TB‐CD) or intervention diet (TB‐ID) (*Figure* S1). Both groups were matched for body weight and tumour volume. When a total tumour volume of 100 mm^3^ was reached or in case of signs of dyspnoea due to tumour growth or pulmonary constriction, humane endpoints were applied.

### Orthotopic lung cancer cachexia (OLCC) mouse model

Murine 344P lung epithelium‐derived adenocarcinoma cells^28^ were cultured with Roswell Park Memorial Institute 1640 medium (Gibco, Rockville, MD, USA) supplemented with 9% fetal bovine serum (Gibco, Rockville, MD, USA). Tumour cells were trypsinized in a sub‐confluent state and suspended in Matrigel matrix (Corning Inc., NY, USA) at a well‐established concentration (2 × 10^6^ cells/mL). Animals were anaesthetised using a mixture of air and isoflurane (4% induction, 2% maintenance) and placed in a position of right lateral decubitus. Fur was removed and a 1 cm superficial skin incision was made below the left scapula. Fat was removed and underlying muscles (excluding intercostal muscles) were carefully lifted. While visualizing the lung motion, 10 μL of Matrigel with or without (sham) tumour cells (2 × 10^4^ cells in 10 μL) were injected through the intercostal space into the lung. The muscles were placed back on top of the rib cage in the correct orientation and the skin was closed using a 5‐0 suture. All mice receive pre‐operative analgesia (Carprofen and Buprenorphine) via subcutaneous injection and post‐operative analgesia (Carprofen) in the drinking water for 2 days.

### Composition of intervention diet

The intervention diet is an AIN‐93M‐based semi‐synthetic modified diet, which is isocaloric to control diet (AIN‐93 M, Bio Services BV, Uden, the Netherlands). The composition of the intervention diet and control diet is shown in *Table* 1. The diet was provided in pellets ad libitum and replaced every 3 days.

**Table 1:** Nutritional composition of control and intervention diet

	Control diet g/kg (EN%)	Intervention diet g/kg (EN%)
Total nutritional value		
Total protein	123 (14%)	216 (24%)
Total fat	41 (10%)	41 (10%)
Total carbohydrates	688 (76%)	592 (66%)
Total energy	3613 kCal	3601 kCal

Raw materials^a^	g/kg	g/kg
Protein sources		
Casein	140	‐
Whey	‐	238
L‐leucine	‐	8.6
L‐isoleucine	‐	2.5
L‐valine	‐	3.5
Fat sources		
Fish oil	‐	25
EPA in fish oil	‐	6.9
DHA in fish oil	‐	3.0
Soybean oil	39	15
Carbohydrates sources		
Corn starch	604	492
Sucrose	97	93
Cellulose (fibre)	48	22
GOS	‐	18
FOS	‐	2
Others/micronutrients		
Mineral mix AIN‐93 M‐MX^b^	35	35
Vitamin mix AIN‐93‐VX^b^	10	10
Additional vitamin D^c^	‐	0.1 (4000 IU)
Choline bitartrate	2.5	2.5
tBHQ	0.008	0.008

DHA, docosahexaenoic acid; EN%, energy percentage; EPA, eicosapentaenoic acid; FOS, fructo‐oligosaccharides; GOS, galacto‐oligosaccharides; kCal, kilocalories; tBHQ, tert‐butylhydroquinone.

^a^ Raw materials sources applied to provide nutrients. Note that these raw materials are food sources that are not 100% pure and bring small amounts of other macro‐ and micronutrients that are taken into account in the total nutritional value calculations of the diets.

^b^ Mineral and vitamin mixes as defined by Reeves et al.^43^

^c^ Total vitamin D is 1000 IU in control diet and 5000 IU in the intervention diet.

### Experimental protocol

A timeline of the experimental protocol is shown in *Figure* S2. All measurements were performed at a standardized time window during their inactive period of the day. Body weight and food intake were measured daily. Food intake was estimated by daily weighing of the pellets that remained in the rack. At baseline and weekly after surgery, grip strength was assessed and μCBCT (Micro Cone Beam Computed Tomography) imaging was performed for all mice to assess lung tumour development^29^ and detect muscle volume changes^30^ over time. At baseline and bi‐weekly after surgery, blood (150 μL) was collected via puncture of the lateral vena saphena. At the end of the experiment, after 5 days of consecutive body weight loss, mice were scanned with a μCBCT scanner and subsequently sacrificed using pentobarbital overdose. Skeletal muscles [e.g., m. soleus, m. plantaris, m. gastrocnemius, m. tibialis anterior (TA) and m. extensor digitorum longus (EDL)] were collected from both hind limbs, using standardized dissection methods. Subsequently, muscles were immediately weighed in pairs on an analytical balance with precision of ±0.1 mg and a linearity of 0.2 mg (CP64, Sartorius, Goettingen, Germany) and snap frozen in liquid nitrogen. The lungs, spleen, epididymal fat, blood, and caecum content were collected and stored for further analysis.

### Grip strength assessment

Grip strength was assessed weekly during the inactive period of the day. Forelimb grip strength was measured with a calibrated grip strength tester (Bioseb, Vitrolles, France) by allowing the mouse to grasp the trapeze bar with both forelimbs and pulling the mouse gently by the tail‐base, parallel to orientation of the meter. For each measurement, a set of five repetitions was performed with 30 s recovery time in between. The mean of the trials was taken as an index of forelimb grip strength.

### Micro cone beam computed tomography

Animals were anaesthetized as outlined in the Animals section, placed in prone position with toes facing the flanks (foot and tibia angle ±90°), and scanned using a μCBCT scanner (X‐RAD 225Cx, Precision X‐Ray Inc., North Branford, USA) at an X‐ray tube potential of 50 kVp and X‐ray tube current of 5.6 mA, as described before.^27^ Deep learning algorithms were used to automatically determine skeletal muscle mass^30^ and tumour volume^29^ on the μCBCT images.

### Fatty acid analysis

Dried blood spot (DBS) sampling was used to collect blood for fatty acid analysis. In short, a drop of blood, obtained from the vena saphena, was collected on a Whatman 903 protein saver card (GE Healthcare Ltd, UK). The samples were air‐dried for 3 h and stored at −25°C in a zip lock foil paper (GE Healthcare Ltd, UK). DBS were analysed by Nutrition Analytical Service at the Institute of Aquaculture, University of Stirling (Stirling, UK). Briefly, a single 5 mm disc was punched out of the DBS. The dried blood was extracted from the spot in 0.1% SDS and methanol at 37°C for 30 min. Following evaporation of the methanol, the samples were resuspended in acetonitrile:ultra‐pure water (1:1) before derivatization using the Waters AccQ‐Tag Ultra Derivatization kit. Amino acid profiles were determined by UPLC instrumentation using the Waters UPLC® Amino Acid Analysis (AAA Solution) on a Waters H‐Class UPLC and an Acquity BEH Phenyl 1.7u UPLC column.

### ELISA assay

Blood was collected in heparin tubes. Blood was centrifuged (2000× *g* for 10 min at 4°C), and plasma was stored at −80°C. Plasma pentraxin‐2 (MPTX20, R&D systems, MN, USA) and CXCL1/KC (MKC00B, R&D systems, MN, USA) levels were quantified using Quantikine ELISA kits according to manufacturers' protocol.

### RNA extraction

For mRNA expression analysis, TRI Reagent (Sigma‐Aldrich, MO, USA) was used according to the manufacturers' protocol. Muscle gastrocnemius was grinded into powder and homogenized in TRI Reagent by using a Mini bead beater (Cole Parmer, IL, USA) sample homogenizer, and total RNA was extracted. A Nanodrop ND‐1000 (Isogen Life Science, De Meern, the Netherlands) was used to measure the quantity and purity of the RNA.

### Reverse transcription quantitative polymerase chain reaction

A fixed amount of 400 ng of total RNA was used for the RT reaction. First‐strand cDNA synthesis was performed using the Tetro cDNA Synthesis Kit according to the manufacturers' protocol (GC‐Biotech, Waddinxveen, the Netherlands). cDNA was diluted (1:50) in nuclease‐free H_2_O and stored at 4°C, cDNA stocks were stored at −20°C. For real‐time PCR amplification, each reaction contained 5 μL SyBr‐green mix (Sensimix SYBR & Fluorescein, GC Biotech), 0.6 μL PCR primer mix (5 μM forward primer + 5 μM reverse primer) and 4.4 μL diluted cDNA template. PCR settings were 95°C for 10 min, followed by 45 cycles of 95°C for 10 s and 60°C for 20 s, carried out on a Roche LightCycler480 system. Melt curves were made using a gradual increase in temperature of 0.11°C/s with five acquisitions/s and a temperature range of 60°C to 90°C. The melt curves were examined using the LightCycler480 software (Roche, Basel, Swiss). PCR efficiency was determined using LinRegPCR software. The resultant N_0_ values were exported and normalized to the geometric average of five reference genes using GeNorm. Cut‐off number of cycles was set as 40. The thresholds used to determine significance of differentially expressed mRNAs was set as *P* < 0.05 and a C_q_ ≥ 1 cycle. The primers used are listed in *Table* S1.

### Western blot

Muscle gastrocnemius was grinded into powder and homogenized in 400 μL whole cell lysate buffer as described before.^27^ The homogenates were incubated for 30 min on ice and centrifuged (14 000× *g*) for 30 min at 4°C. Subsequently, supernatant fraction was separated from the pellet fraction. Protein concentration of the fractions was determined by using a BCA protein assay kit (Pierce, Thermo Fischer Scientific, MA, USA).

For western blot analysis, Laemmli buffer (0.25 M Tris–HCl pH 6.8, 8% (*w*/*v*) SDS, 40% (*v*/*v*) glycerol, 0.4 M DTT and 0.02% (*w/v*) Bromophenol blue) was added in a 1:4 dilution to the supernatant fraction and were incubated for 5 min at 95°C. Equal amounts of protein were loaded per lane on a Criterion XT Precast 4–12% Bis‐Tris gel (Bio‐Rad, CA, USA). Proteins were separated by electrophoresis (100–140 V). The proteins were transferred to a 0.45 μm nitrocellulose membranes (Bio‐Rad, CA, USA) by electroblotting (Bio‐Rad Criterion Blotter) at 100 V for 1 h. Membranes were stained with Ponceau S (0.2% Ponceau S in 1% acetic acid; Sigma‐Aldrich, MO, USA) to quantify total protein loading. Subsequently, membranes were washed with Tris‐buffered saline with 0.05% Tween‐20 (TBS‐T) and blocked for 1 h at room temperature in 5% non‐fat dried milk (ELK, Campina, Amersfoort, the Netherlands) diluted in TBS‐T. Membranes were washed with TBS‐T and overnight incubated at 4°C with specific primary antibodies (1:1000). Subsequently, membranes were washed with TBS‐T and were incubated with secondary antibodies (1:5000) of anti‐mouse IgG peroxidase (Vector) or anti‐rabbit peroxidase (Vector). Detection was performed using SuperSignal West Pico Chemiluminescent substrate (Thermo Fischer Scientific, MA, USA) according to the manufacturers' protocol. Membranes were imaged (Amersham Imager 600, GE Life Sciences, NJ, USA) and quantified using the ImageQuantTL software (GE life sciences, NJ, USA). The intensity of the bands of the proteins of interest were corrected for total protein loading assessed by Ponceau S staining. The primary antibodies used are listed in *Table* S2.

### Statistics

All data is presented as mean ± standard error of the mean (SEM). Repeated measures were analysed by mixed models with post hoc Fisher's least significant difference. Comparisons between two variables were statistically tested with the two‐tailed unpaired *t*‐test for normally distributed data or Mann–Whitney *U* test for non‐parametric data. Comparisons between three or more variables were statistically tested with the one‐way ANOVA and Bonferroni's multiple comparison post hoc test. Statistical analysis was performed using IBM SPSS Statistics 25. Significance was set at *P* < 0.05.

## Results

### Targeted intervention diet delays development and attenuates progression of cachexia

**Figure 1:**
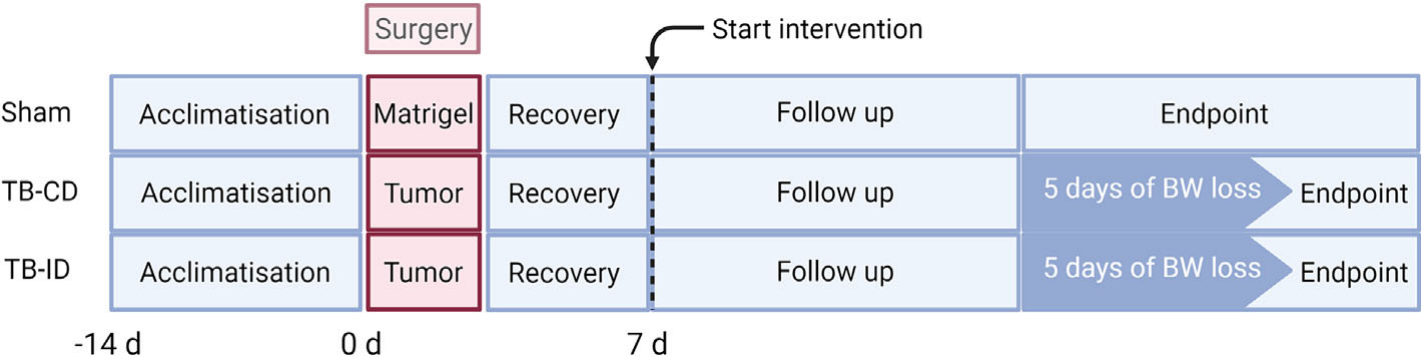
Experimental outline.

In order to determine the therapeutic effect of the intervention diet, tumour‐bearing mice were stratified to either control diet (TB‐CD) group or intervention diet (TB‐ID) group and evaluated for development of cachexia. Drop‐outs, tumour‐bearing mice that reached predefined humane endpoint criteria before they developed cachexia, were not significantly different between TB‐CD and TB‐ID (28% vs. 35% respectively, excluded from further analyses). The remaining mice of the TB‐CD group and TB‐ID group were followed‐up until they had developed the cachexia endpoint, predefined as five consecutive days of body weight loss (*Figure* 1).

**Figure 2:**
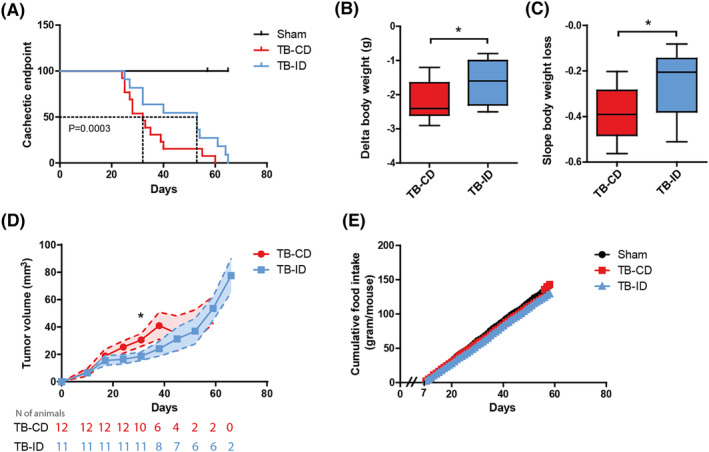
Intervention diet delays the onset and attenuates the progression of body weight loss. (A) Kaplan–Meier plot was constructed to assess the average time until cachexia endpoint (based on predefined experimental endpoint) of tumour‐bearing mice on control diet (TB‐CD) versus intervention diet (TB‐ID). TB‐CD mice developed cachexia significant earlier when compared with TB‐ID mice (log‐rank *P*‐value: 0.0003). (B) Body weight loss during the last 5 days of the experiment. (C) Slope of the linear curve of the body weight change during the last 5 days of the experiment. (D) Tumour volume (mm^3^) in TB‐CD and TB‐ID mice over time, measured by μCBCT. (E) Cumulative food intake from 7 days post‐surgery. All data are presented as mean ± SEM, sham control *n* = 9, TB‐CD *n* = 12, and TB‐ID *n* = 11. Repeated measures were statistically tested using mixed model analysis. Comparisons were statistically tested with the two‐tailed unpaired *t*‐test for normally distributed data or Mann–Whitney *U* test for non‐parametric data. Significance is shown as *P < 0.05.

Survival analysis performed on all experimental groups (sham *n* = 9, TB‐CD *n* = 12, and TB‐ID *n* = 11) revealed a significant delay in the average time until the cachectic endpoint in the TB‐ID group compared with the TB‐CD group (46.0 ± 15.2 days vs. 34.7 ± 11.4 days, *P* = 0.0003; *Figure* 2A). In addition, the body weight loss during the last 5 days of the experiment was analysed. The amount (−1.57 ± 0.62 vs. −2.13 ± 0.57 g, *P* = 0.031; *Figure* 2B) and progression (−0.26 ± 0.14 vs. −0.39 ± 0.11, *P* = 0.024; *Figure* 2C) of body weight loss was significantly reduced in the TB‐ID group compared with the TB‐CD group. The intervention diet tends to inhibit tumour growth. However, this only became significant at day 31 of the experiment (*Figure* 2D).

**Figure 3:**
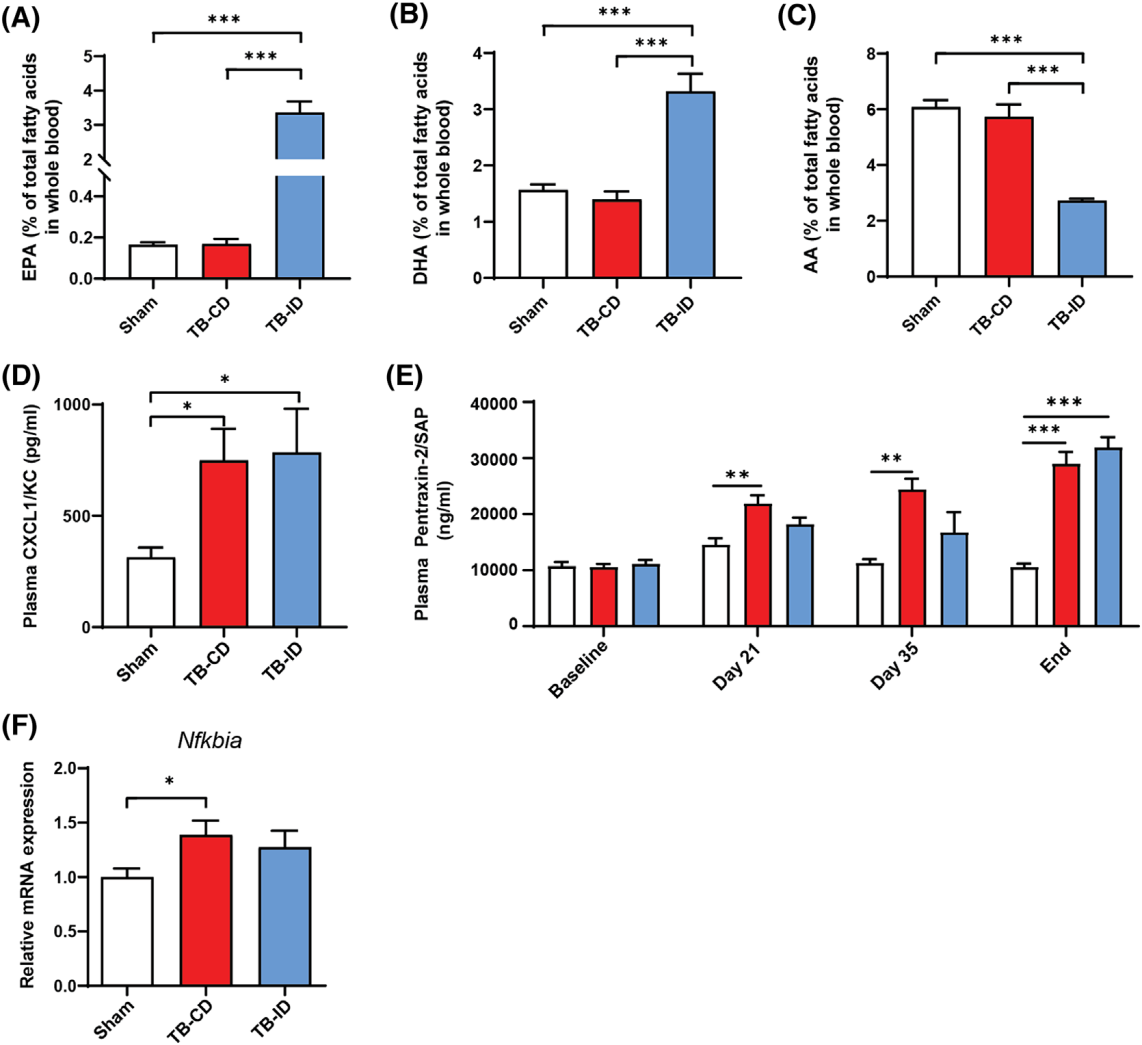
Intervention diet decreases n‐6/n‐3 lcPUFA ratio in whole blood and suppresses systemic inflammation. (A) EPA, (B) DHA, and (C) AA levels presented as percentage of total fatty acids in whole blood. (D) CXCL1/KC (pg/mL) and (E) Pentraxin‐2 (ng/mL) expression levels in blood plasma. (F) Relative mRNA expression of Nfkbia (IкBα) measured in the m. gastrocnemius. All data are presented as mean ± SEM. Comparisons were statistically tested with the one‐way ANOVA and Bonferroni's multiple comparison post hoc test. Significances are shown as *P < 0.05, **P < 0.01, and ***P < 0.001.

No differences in food intake were observed between the groups over time (*Figure* 2E). To investigate the systemic effects of the intervention diet, fatty acid abundance and markers for inflammation were assessed in blood at the end of the study. The intervention diet, which is rich in n‐3 lcPUFAs, caused a significant increase in both EPA and DHA, and a decrease in arachidonic acid (AA) blood levels compared with sham control and control‐fed TB mice (*Figure* 3A–C). As markers for systemic inflammation, plasma levels of the pro‐inflammatory chemokine CXCL1/KC (murine IL‐8 orthologue) and acute phase protein pentraxin‐2 were assessed. CXCL1/KC and pentraxin‐2 plasma levels were similarly increased in TB‐CD and TB‐ID mice compared with sham control mice at the cachectic endpoint of the study (*Figure* 3D,E). However, before cachexia was evident, significant increases in pentraxin‐2 plasma levels were detectable in TB‐CD but not TB‐ID mice compared with control [day 21 (*P* = 0.003) and day 35 (*P* = 0.007), *Figure* 3E]. In addition, the mRNA expression of *Nfkbia* (IκBα) was increased in muscle of TB‐CD mice compared with sham control mice (*P* < 0.05); *Figure* 3F), but not in the muscle of TB‐ID mice. This suggests anti‐inflammatory properties of the intervention diet.

**Figure 4:**
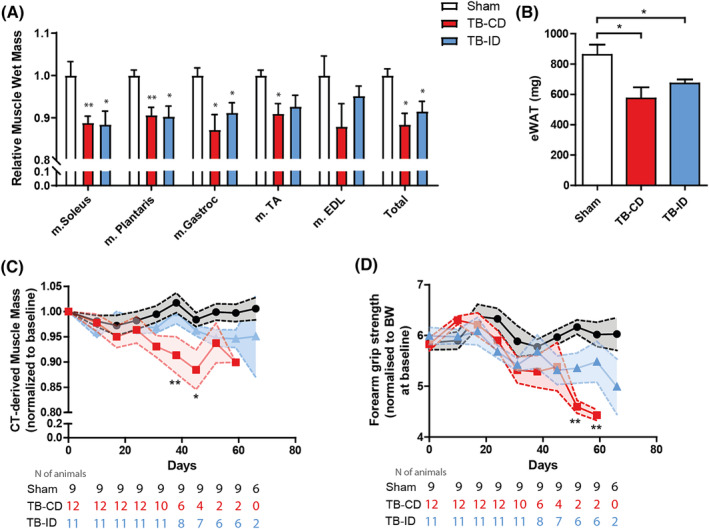
Loss of muscle mass, epididymal white adipose tissue and muscle function in tumour‐bearing mice. (A) Relative change in muscle wet mass at the cachexia endpoint of the experiment. (B) eWAT weight at the cachexia endpoint of the experiment. (C) μCBCT‐derived muscle mass over time, normalized to μCBCT‐derived muscle mass at baseline (pre‐surgery). (D) Forearm grip strength (gram) over time, normalized to total body weight (gram) at baseline. Comparisons in panels (A) and (B) were statistically tested with the one‐way ANOVA and Bonferroni*'*s multiple comparison post hoc test. Repeated measures in panels (C) and (D) were statistically tested using mixed model analysis. Significant differences between sham and TB‐CD are shown as *P < 0.05 and ^**^ *P* < 0.001.

The development of cachexia in the TB‐CD and TB‐ID groups was characterized by a significant reduction in skeletal muscle mass (*Figure* 4A) and epididymal white adipose tissue (eWAT) (*Figure* 4B) compared with sham control. No differences in these endpoint measurements were observed between the TB‐CD group and the TB‐ID group at the predefined cachexia endpoint. The loss of muscle mass was associated with significant loss of muscle function in both the TB‐CD and TB‐ID group (*Figure* S3). Interestingly, longitudinal examination of lower hind limb muscle mass through μCBCT (*Figure* 4C) and concurrent assessment of muscle function (*Figure* 4D) revealed that the delay in the development of cachexia is accompanied by prolonged maintenance of both muscle mass and function in the TB‐ID group.

Combined, these findings demonstrate that the intervention diet delays the development of cachexia, reduces the progression of body weight loss and attenuates systemic inflammation and loss of muscle mass and function during the development of cachexia in mice with lung cancer.

### Targeted intervention diet attenuates activation of proteolysis signalling in the muscle of tumour‐bearing mice

**Figure 5:**
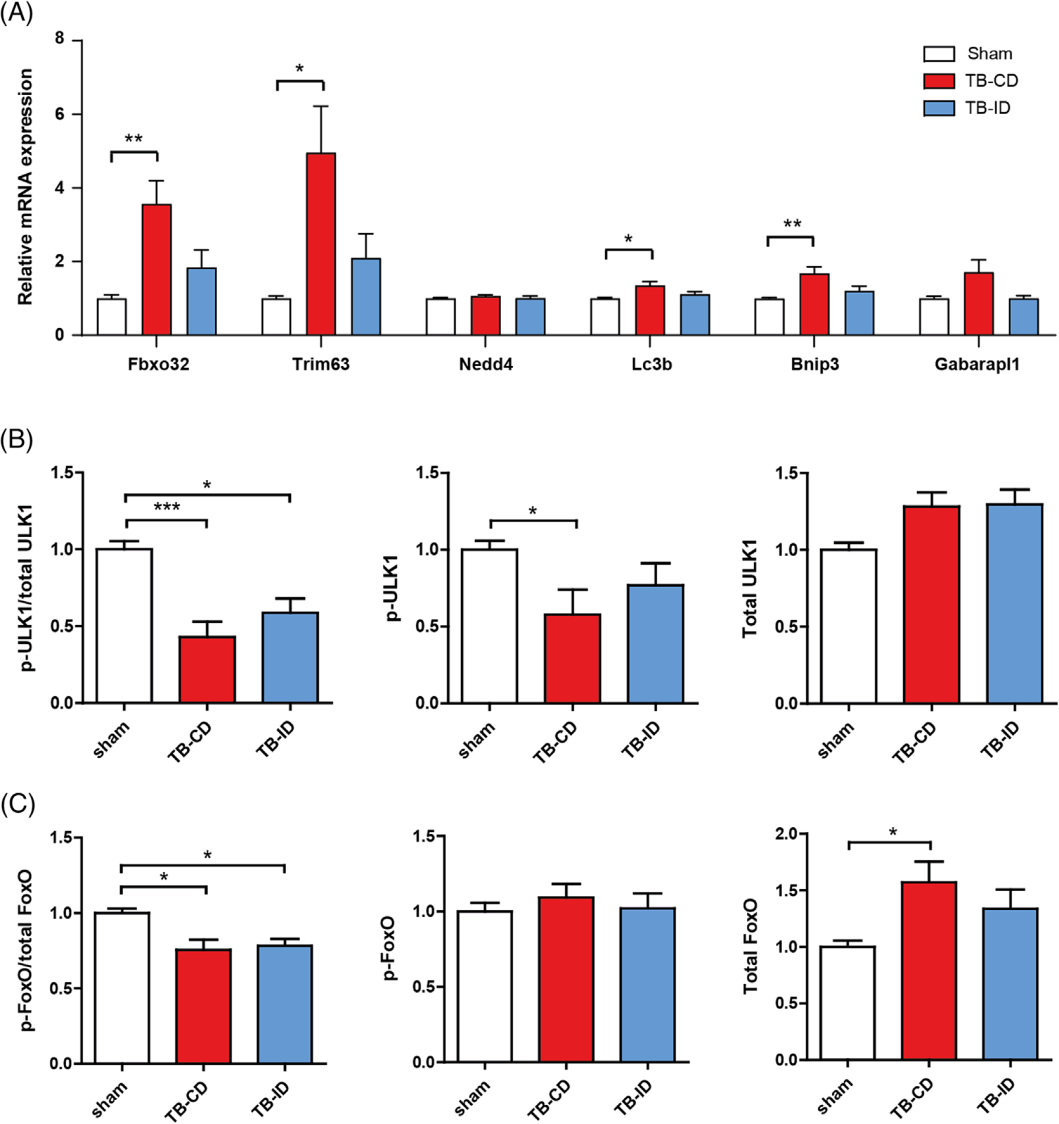
Intervention diet attenuates activation of proteolysis signalling in the muscle of tumour‐bearing mice. (A) Relative mRNA expression of the E3 ligases: Fbxo32, Trim63, and Nedd4; and of components of the autophagy lysosomal pathway: Lc3b, Bnip3, and Gabarapl1. (B) Ratio of phosphorylated ULK1 (Ser757) over total ULK1, and the relative protein expression of phosphorylated ULK1 (Ser757) and total ULK1. (C) Ratio of phosphorylated FoxO1 (Ser256) over total FoxO1, and the relative protein expression of phosphorylated FoxO1 (Ser256) and total FoxO1. Representative pictures of the Western blot data are shown in *Figure* S4. All data are presented as mean ± SEM, sham control *n* = 9, TB‐CD *n* = 12, and TB‐ID *n* = 11. Comparisons were statistically tested with the one‐way ANOVA and Bonferroni*'*s multiple comparison post hoc test. Significances are shown as *P < 0.05, **P < 0.01, and ***P < 0.001.

The ubiquitin proteasome system (UPS) and autophagy lysosomal pathway (ALP) are the most important pathways for protein degradation in skeletal muscle.^31^, ^32^ To investigate the anti‐catabolic effect of the intervention diet, mRNA and protein expression levels of critical mediators of both the UPS and ALP were assessed. When compared with the sham control group, the mRNA expression of *Fbxo32*, *Trim63*, *Lc3b*, and *Bnip3* were upregulated in the TB‐CD group, but not in the TB‐ID group (*Figure* 5A). The protein levels of phosphorylated ULK1 over total ULK1 were decreased in the TB‐CD and TB‐ID groups compared with the sham control group (*Figure* 5B). In the TB‐CD group, this was mainly due to decreased levels of phosphorylated ULK1. Finally, the protein levels of phosphorylated FoxO1 over total FoxO1 were reduced in the TB‐CD and TB‐ID groups compared with the sham control group, mainly by increased levels of total FoxO1 (*Figure* 5C). Altogether, these findings suggest that the intervention diet attenuated full activation of proteolysis signalling in the muscle of tumour‐bearing mice with cachexia.

### Targeted intervention diet modulates protein synthesis signalling in the muscle of tumour‐bearing mice

**Figure 6:**
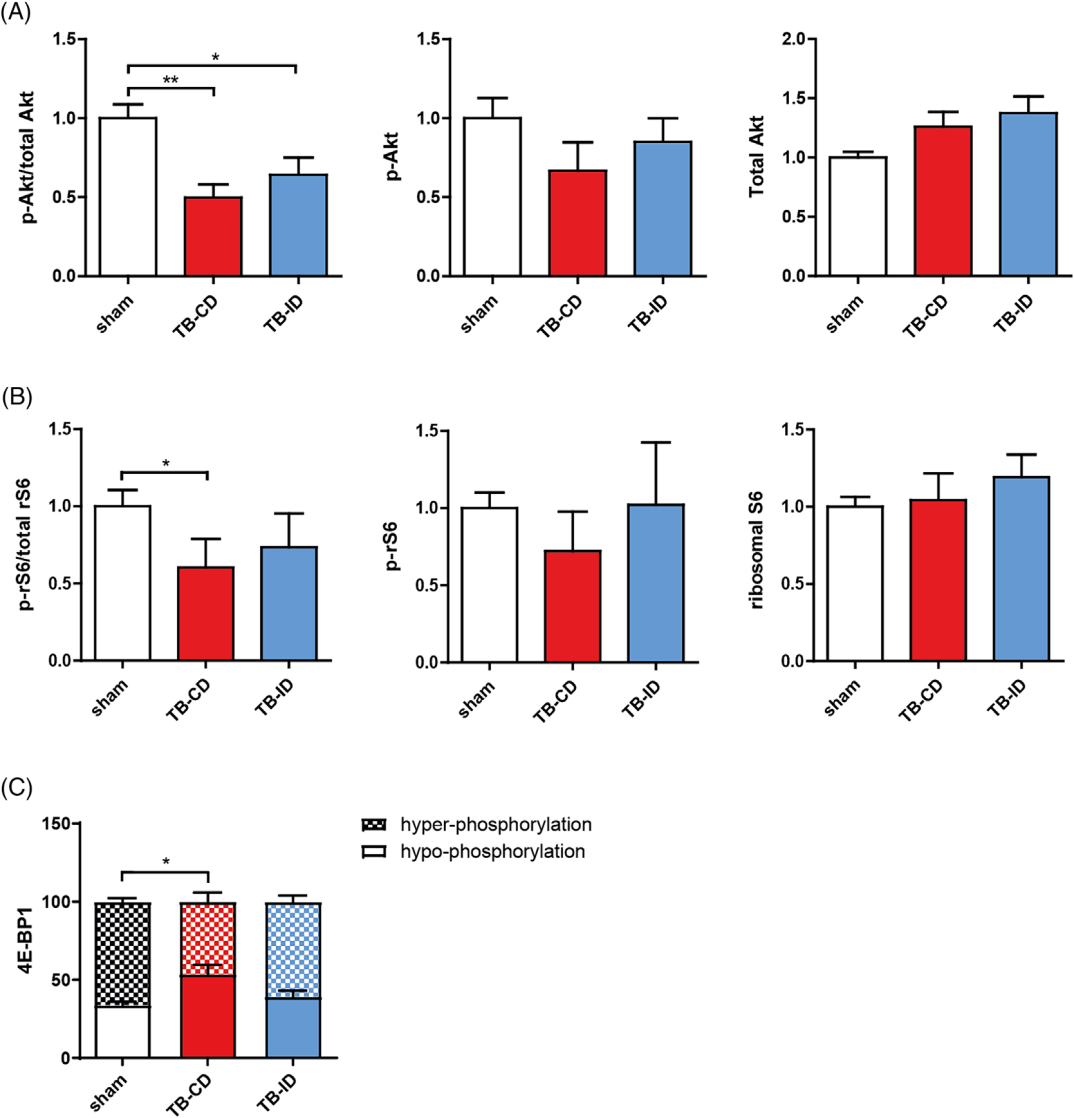
Intervention diet preserves protein synthesis signalling in the muscle of tumour‐bearing mice. (A) Ratio of phosphorylated Akt (Ser473) over total Akt, and the relative protein expression of phosphorylated Akt (Ser473) and total Akt. (B) Ratio of phosphorylated S6 (Ser235/236) over total S6, and the relative protein expression of phosphorylated S6 (Ser235/236) and total S6. (C) Phosphorylation distribution of 4E‐BP1, hypo‐phosphorylated 4E‐BP1, and hyper‐phosphorylated 4E‐BP1 (grid). Representative pictures of the western blot data are shown in *Figure* S4. All data are presented as mean ± SEM, sham control *n* = 9, TB‐CD *n* = 12, and TB‐ID *n* = 11. Comparisons were statistically tested with the one‐way ANOVA and Bonferroni*'*s multiple comparison post hoc test. Significances are shown as *P < 0.05 and **P < 0.01.

To assess the potential anabolic effects of the intervention diet, the activity of multiple components of the insulin growth factor 1 (IGF‐1) signalling pathway were measured. When compared with the sham control group, the phosphorylation of Akt was reduced in the TB‐CD and TB‐ID groups (*Figure* 6A). Downstream of Akt, the activation of ribosomal S6 was decreased in the TB‐CD group compared with sham control (*Figure* 6B). In addition, when compared with control, a shift from mainly hyper‐to hypo‐phosphorylated 4E‐BP1 was present in the TB‐CD group (*Figure* 6C). These reductions in phosphorylation levels of ribosomal S6 and 4E‐BP1 were not observed in the TB‐ID group. Combined, these findings indicate that the intervention diet modulates the decline in some but not all components of protein synthesis signalling in the skeletal muscle of tumour‐bearing mice with cachexia.

## Discussion

In this study, we showed for the first time that an intervention diet with a specific nutritional combination of high protein (100% whey protein), L‐leucine, vitamin D, fish oil, GOS, and FOS delays the development of experimental lung cancer cachexia and modulates cachexia associated metabolic alterations. This intervention diet extends the time until development of cachexia and attenuates the progression of body weight‐ and muscle mass loss, resulting in prolonged maintenance of muscle function. These beneficial properties of the intervention diet are accompanied by modulatory effects on systemic inflammation and muscle protein turnover.

Cancer cachexia is recognized as a predictor of poor clinical outcome, decreased survival, and limited efficacy of cancer treatment.^4^ In addition, muscle wasting is an important contributing factor to muscle weakness in cachexia, which adversely affects performance status, quality of life and hospitalization risk of cancer patients. In this study, we tested the therapeutic effect of a multinutrient intervention diet on the development of cancer cachexia in an orthotopic lung cancer mouse model. We showed for the first time that an intervention diet delays the cachexia endpoint and progression of body weight loss. Importantly, the multinutrient intervention diet does not increase tumour volume despite the fact that it contains nutrients that are known for their anabolic effects on muscle protein synthesis. Our findings in the orthotopic lung cancer mouse model extend observations in previous studies in which C26 tumour‐bearing mice were treated with a similar multinutrient diet, referred to as specific nutritional combination (SNC) diet. The main difference between the intervention diet and SNC diet is the source of protein (whey vs. casein) and the presence of vitamin D. In C26 tumour‐bearing mice, the SNC diet significantly improved body weight, eWAT and muscle mass compared with tumour‐bearing control.^33^, ^34^ In contrast, in the current study we did not observe significant effects of the intervention diet on eWAT and muscle wet mass. This discrepancy can be attributed to differences in the experimental design. The intervention studies in the C26 tumour‐bearing mice were stopped at a fixed time point (day 20 after tumour inoculation), while in this study we continued until the mice developed cachexia. Unique for our study is the longitudinal assessment of muscle mass by μCBCT, which provides additional information on the dynamics and the timing of muscle mass loss. The longitudinal assessment of the muscle mass and concurrent assessment of muscle function indicates that the delayed cachexia endpoint is accompanied by a prolonged maintenance of muscle mass and function in the intervention group. Previously, Norren et al. showed that muscle function (measured ex vivo) was improved and total daily activity was normalized after intervention with the SNC diet.^34^ In oesophageal cancer patients, a 4‐week intervention study showed that the SNC diet significantly improved body weight and EOCG performance status compared with routine care.^35^ Altogether these findings provide (pre)clinical evidence that the multitarget, multinutrient intervention diet positively modulates the cachectic endpoint and progression of cachexia. This is highly relevant as a delayed onset and progression of cachexia potentially provides increased systemic tolerance to cancer treatment and consequently prolonged survival.

Systemic inflammation is a hallmark of cachexia.^4^ In general, cancer patients with cachexia have higher levels of systemic inflammation than those without cachexia.^36^ In accordance with this, we found an inverse correlation between muscle mass and the plasma levels of pentraxin‐2 (data not shown), an acute‐phase protein. Excessive amounts of n‐6 lcPUFA or a very high n‐6/n‐3 ratio promote systemic inflammation and the pathogenesis of many diseases including cancer, whereas increased levels of n‐3 lcPUFA (or a low n‐6/n‐3 ratio) exert suppressive effects. In this study, we show that the intervention diet significantly decreases the n‐6/n‐3 ratio in blood compared with control fed mice. Various studies have evaluated the efficacy of n‐3 lcPUFAs or prebiotic non‐digestible oligosaccharides on cancer cachexia, predicting positive anti‐inflammatory properties. The intervention diet deployed in our study containing fish oil and GOS/FOS seems to partially suppress systemic inflammation, measured by pentraxin‐2 expression levels, during lung tumour development. However, these potential anti‐inflammatory effects of the intervention diet were no longer evident once the mice developed cachexia. As the average time to cachectic endpoint was significantly extended by the intervention diet, these findings suggest that this might be attributable to a direct effect of the intervention diet on systemic inflammation. In support of our findings, the SNC diet significantly reduced plasma levels of pro‐inflammatory cytokines, including interleukin‐6, tumour necrosis factor‐α and prostaglandin E2, in C26 tumour‐bearing mice.^33^ Moreover, in cancer patients, serum PGE_2_ levels were significantly reduced after intervention with the SNC diet.^35^ All together, these findings suggest anti‐inflammatory properties of the multitarget, multinutrient intervention diet that precede the development of cachexia.

In cancer cachexia, the continuous loss of muscle mass is characterized by a negative protein balance.^4^ The skeletal muscle wasting observed in OLCC mice is linked to increased proteolysis, encompassing both UPS and ALP, and decreased protein synthesis. In this study, we show that the intervention diet attenuated alterations in catabolic and anabolic signalling pathways in tumour‐bearing animals compared with control diet. It is however important to note that these mice had also developed cachexia at the time of tissue collection, but that the partial suppression of muscle atrophy signalling is in line with the slower progression of cachexia in response to intervention diet. It is suggested that the intervention diet could modulate protein turnover either directly or indirectly via systemic inflammation. Consistent with the partially suppressed systemic inflammation observed prior to the onset of cachexia, we identified attenuated mRNA expression of IκBα in the skeletal muscle of tumour‐bearing mice subjected to the intervention diet. Given that IκBα serves a target gene of the *NF‐κB* transcription factor, this finding implies reduced inflammatory signalling in the muscle of these mice. This aligns with the partially attenuated *Trim63/MuRF‐1* mRNA expression, as it is suggested that muscle atrophy induced by the constitutive activation of muscle *NF‐κB* relies on the increased expression of *Trim63/MuRF‐1*.^37^ The direct effects on protein turnover could be attributable to the high levels of BCAAs in the intervention diet, which have the ability to activate mTORC1 and its downstream phosphorylation of S6k and 4E‐BP1.^38^ Indeed, in rats bearing the Walker 256 tumour, multiple studies have demonstrated that L‐leucine supplementation did attenuate muscle protein loss, by modulating protein synthesis.^39^, ^40^ Moreover, in cancer patients, the SNC diet increased plasma leucine levels and was able to stimulate muscle protein synthesis.^14^ Although the current experimental setup did not allow us to demonstrate a direct effect of the diet on protein turnover, the observed attenuated alterations in gene expression and signalling alterations strongly indicate a potential modulatory effect that warrants further investigation.

It is important to note that not only tumour activity and host responses may drive cachexia but also the treatments directed at the tumour (i.e., radio‐ and/or chemotherapy) can accelerate or even induce cachexia directly. Consequently, therapies targeting cancer‐associated cachexia may require further specification based on the underlying pathological mechanisms driving tumour‐ or treatment‐induced cachexia. Furthermore, in clinical practice, cancer cachexia will not be treated in isolation, but parallel to the cancer treatment. Therefore, it is important to investigate if therapies targeting cachexia are safe and effective when combined with cancer treatment, and whether they modulate the therapeutic effectiveness. Although not studied in the current proof of concept study, Wijler et al. showed that the multinutrient intervention diet improves muscle contraction capacity and physical activity of chemotherapy‐treated C26 tumour‐bearing mice without affecting treatment efficacy.^41^ However, carefully designed preclinical studies using orthotopic preclinical models will be required to dissect tumour and treatment‐induced mechanisms driving cachexia and to detail the optimal multinutrient composition for safe and effective application of cachexia therapies in the cancer disease management trajectory.

## Conclusions

This study shows that an intervention diet with a specific nutritional combination of high protein (100% whey protein), L‐leucine, vitamin D, fish oil, GOS, and FOS delayed the cachectic endpoint and attenuates progression of cachexia in an orthotopic lung cancer mouse model, resulting in prolonged maintenance of muscle function. Therefore, multinutrient intervention strategies that simultaneously target multiple components of cachexia have high potential value as integral part of lung cancer management.

## Funding

This research was funded as part of a grant of Danone Nutricia Research, grant name “Preclinical development of intervention strategies in lung cancer cachexia”.

## Conflict of interest

Wouter van de Worp has received funds paid to the institution from Danone Nutricia Research. Frank Verhaegen is co‐founder of SmART Scientific Solutions B.V. Ardy van Helvoort is employed by Danone Nutricia Research. The other authors declare no potential conflicts of interest.

## Supporting information


**Figure S1.** Flowchart of the animal experiment. Male mice, 11‐weeks old, were randomly allocated to either sham operated group (n = 9) or tumour‐bearing group (n = 42). Seven days post tumour inoculation, tumours were scored for location and size using the Micro Cone Beam Computed Tomography (μCBCT). If the primary tumour did not appear in the lung, mice were excluded (n = 8). TB mice (n = 34) were allocated to either control diet (TB‐CD, n = 17) or intervention diet (TB‐ID, n = 17). Both groups were matched for body weight and tumour volume. Only the tumour‐bearing mice which developed cachexia (5 days of consecutive bodyweight loss) were included for further analysis.
**Figure S2.** Experimental protocol. Body weight and food intake were measured daily. At baseline (T = ‐3) and weekly after surgery (T = 0), grip strength was assessed and μCBCT (Micro Cone Beam Computed Tomography) imaging was performed for all mice to assess lung tumour development [29] and detect muscle volume changes [30] over time. At baseline and bi‐weekly after surgery, blood (150 μl) was collected via puncture of the lateral vena saphena. At T = 7, TB mice were allocated to either control diet or intervention diet. At the end of the experiment, after five days of consecutive body weight loss, mice were sacrificed using pentobarbital overdose and tissue was collected.
**Figure S3.** The development of cachexia is associated with loss of muscle function in both the TB‐CD and TB‐ID group. Pre: muscle function before start intervention, Post: muscle function at the end of the experiment (cachexia).
**Figure S4.** Representative images of Western Blot data. S: sham, CD: tumour bearing mice on control diet; ID: tumour‐bearing mice on intervention diet. The intensity of the bands was normalized to Ponceau S
**Table S1.** List of primers.
**Table S2.** List of primary antibodies.

## Data Availability

All data are available in the main text or the supplementary materials.
