# Supplementary material for: Targeted nutritional intervention attenuates experimental lung cancer cachexia
Source: J Cachexia Sarcopenia Muscle. 2024 Jul 4;15(5):1664–76. doi: 10.1002/jcsm.13520 (PMC11446694; doi:10.1002/jcsm.13520)
Supplement: Supplementary file 1 — Figure S1. Flowchart of the animal experiment. Male mice, 11‐weeks old, were randomly allocated to either sham operated group (n = 9) or tumour‐bearing group (n = 42). Seven days post tumour inoculation, tumours were scored for location and size using the Micro Cone Beam Computed Tomography (μCBCT). If the primary tumour did not appear in the lung, mice were excluded (n = 8). TB mice (n = 34) were allocated to either control diet (TB‐CD, n = 17) or intervention diet (TB‐ID, n = 17). Both groups were matched for body weight and tumour volume. Only the tumour‐bearing mice which developed cachexia (5 days of consecutive bodyweight loss) were included for further analysis. Figure S2. Experimental protocol. Body weight and food intake were measured daily. At baseline (T = ‐3) and weekly after surgery (T = 0), grip strength was assessed and μCBCT (Micro Cone Beam Computed Tomography) imaging was performed for all mice to assess lung tumour development [29] and detect muscle volume changes [30] over time. At baseline and bi‐weekly after surgery, blood (150 μl) was collected via puncture of the lateral vena saphena. At T = 7, TB mice were allocated to either control diet or intervention diet. At the end of the experiment, after five days of consecutive body weight loss, mice were sacrificed using pentobarbital overdose and tissue was collected. Figure S3. The development of cachexia is associated with loss of muscle function in both the TB‐CD and TB‐ID group. Pre: muscle function before start intervention, Post: muscle function at the end of the experiment (cachexia). Figure S4. Representative images of Western Blot data. S: sham, CD: tumour bearing mice on control diet; ID: tumour‐bearing mice on intervention diet. The intensity of the bands was normalized to Ponceau S Table S1. List of primers. Table S2. List of primary antibodies. [file JCSM-15-1664-s001.docx]

**Supplementary Data**


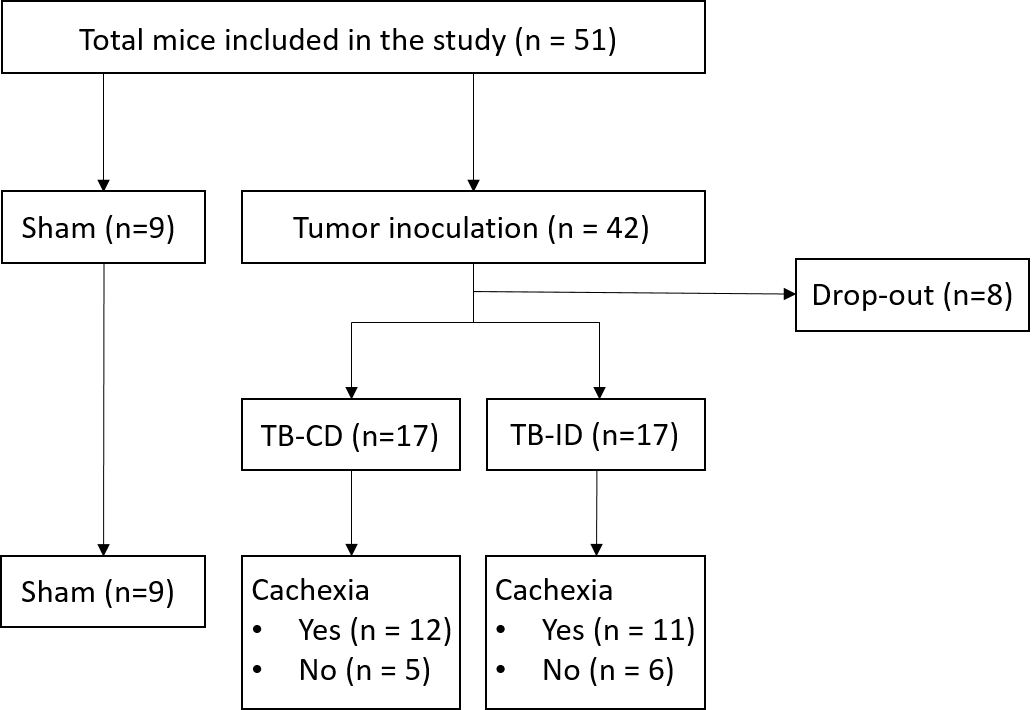


***Fig. S1: Flowchart of the animal experiment.*** *Male mice, 11-weeks old, were randomly allocated to either sham operated group (n=9) or tumor-bearing group (n=42). Seven days post tumor inoculation, tumors were scored for location and size using the Micro Cone Beam Computed Tomography (µCBCT). If the primary tumor did not appear in the lung, mice were excluded (n=8). TB mice (n=34) were allocated to either control diet (TB-CD, n=17) or intervention diet (TB-ID, n=17). Both groups were matched for body weight and tumor volume. Only the tumor-bearing mice which developed cachexia (5 days of consecutive bodyweight loss) were included for further analysis.*


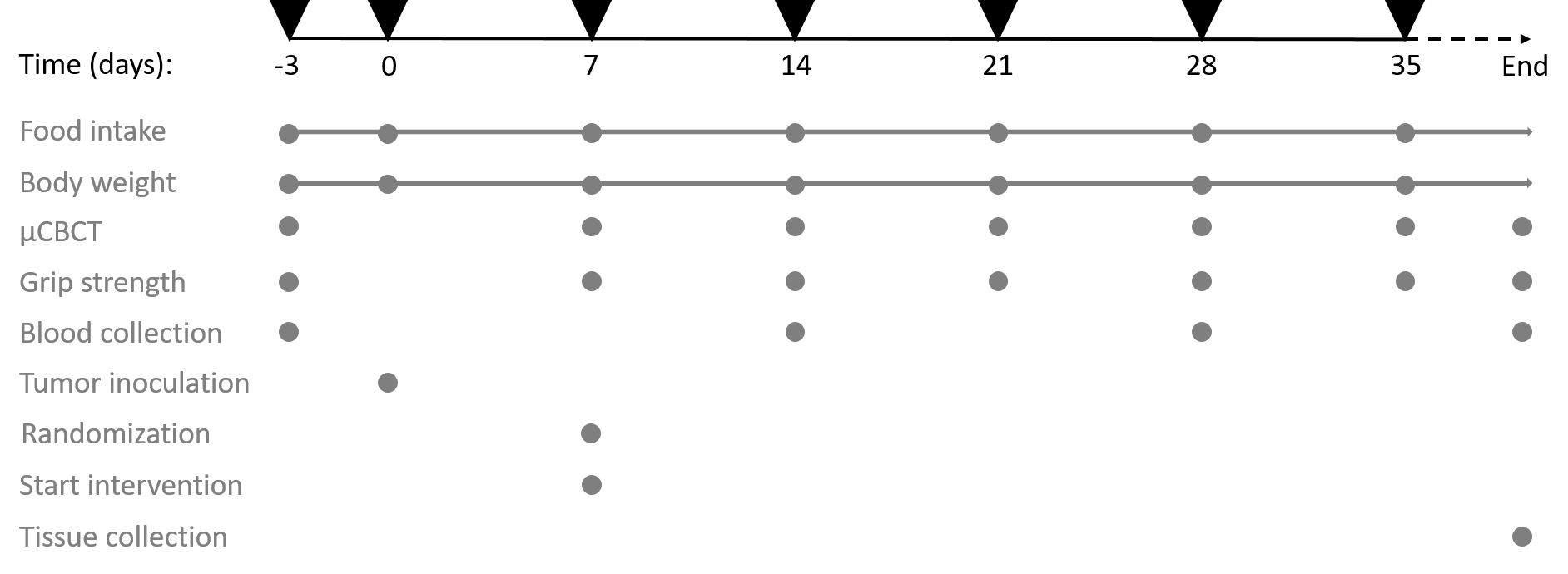


***Fig. S2: Experimental protocol.*** *Body weight and food intake were measured daily. At baseline (T=-3) and weekly after surgery (T=0), grip strength was assessed and µCBCT (Micro Cone Beam Computed Tomography) imaging was performed for all mice to assess lung tumor development [29] and detect muscle volume changes [30] over time. At baseline and bi-weekly after surgery, blood (150 µl) was collected via puncture of the lateral vena saphena. At T=7, TB mice were allocated to either control diet or intervention diet. At the end of the experiment, after five days of consecutive body weight loss, mice were sacrificed using pentobarbital overdose and tissue was collected.*


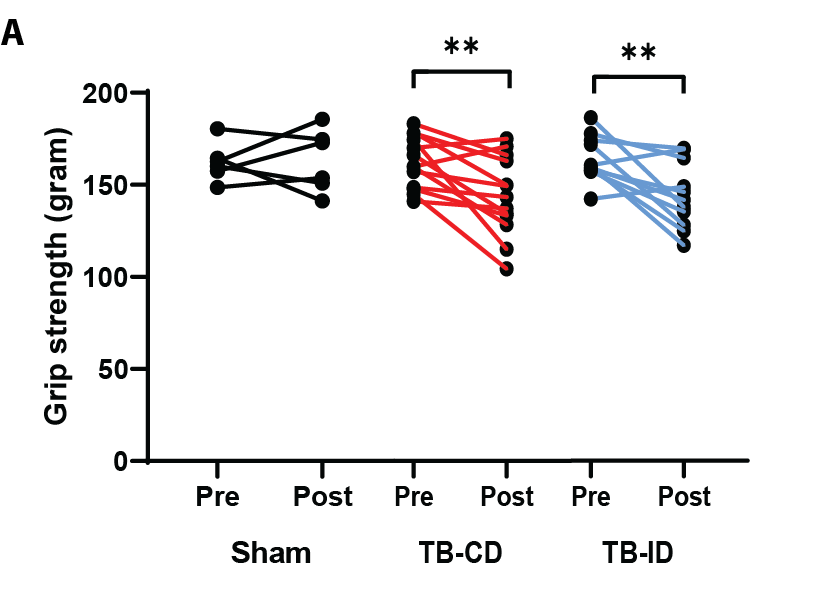


***Fig. S3: The development of cachexia is associated with loss of muscle function in both the TB-CD and TB-ID group. Pre: muscle function before start intervention, Post: muscle function at the end of the experiment (cachexia).***


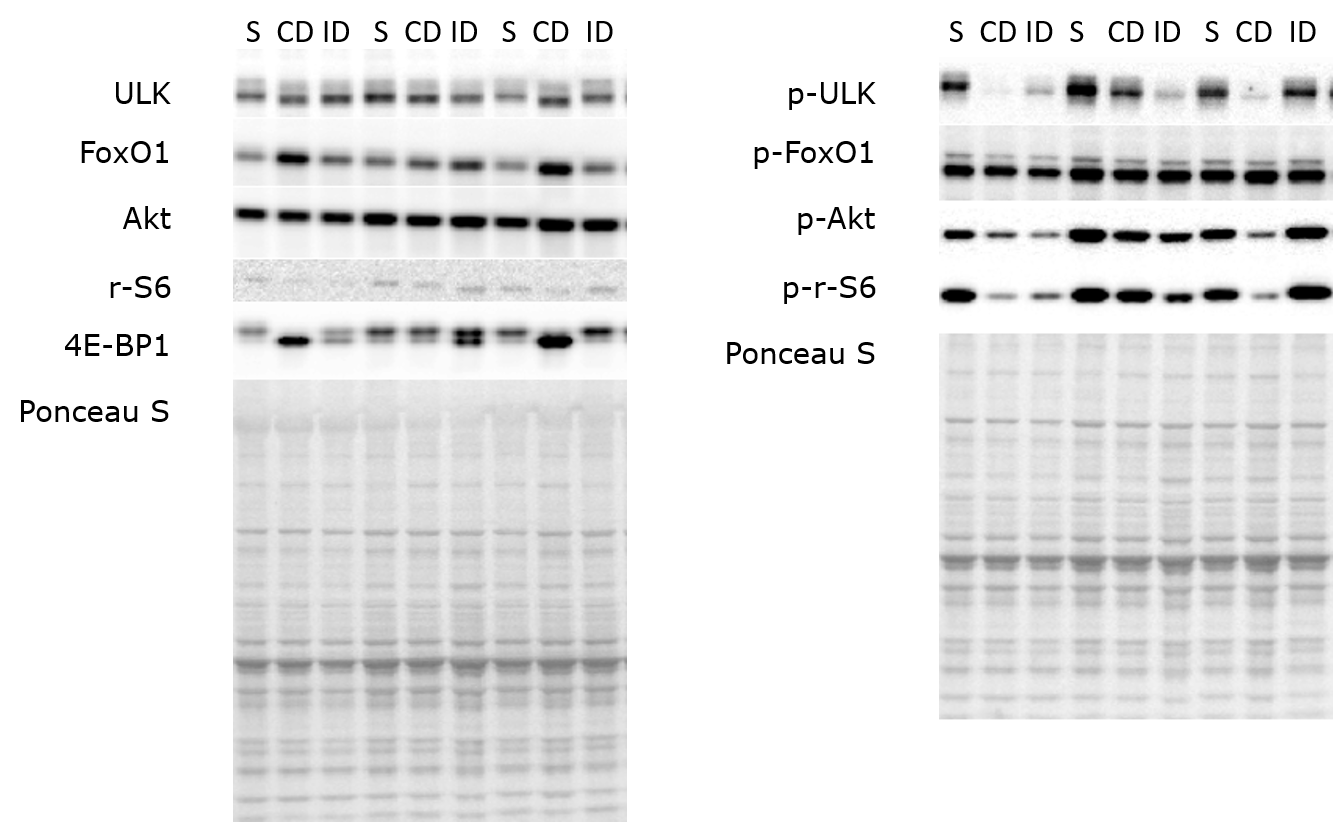


***Fig. S4: Representative images of Western Blot data****. S: sham, CD: tumor bearing mice on control diet; ID: tumor-bearing mice on intervention diet. The intensity of the bands was normalized to Ponceau S*

**Table S1: List of primers.**

| **Gene name** | **Forward primer** | **Reverse primer** |
| --- | --- | --- |
| *Fbxo32* | CAGCAGCTGAATAGCATCCAGAT | TCTGCATGATGTTCAGTTGTAAGC |
| *Trim63* | CTTCCTCTCAAGTGCCAAGCA | GTGTTCTAAGTCCAGAGTAAAGTAGTCCAT |
| *Nedd4* | GCTGCCAAGAGCACACACCTG | CAACGCCATCAAAGCCCTGT |
| *Bnip3* | AGGTTTTCCTTCCATCTCTGTTACTG | TGTGTGAACAGAAGTCAGATCCAAA |
| *Gabarapl1* | CCCTCCCACCAGTGCTACCAT | TCATCACTGTAGGCCACATACAGAAAA |
| *Lc3b* | GAGCAGCACCCCACCAAGAT | CGTGGTCAGGCACCAGGAA |
| *Nfkbia* | GCTACCCGAGAGCGAGGAT | GCCTCCAAACACACAGTCATCA |
| *Housekeeping genes:* | | |
| *Rplp0* | GGACCCGAGAAGACCTCCTT | GCACATCACTCAGAATTTCAATGG |
| *Rpl13a* | CACTCTGGAGGAGAAACGGAAGG | GCAGGCATGAGGCAAACAGTC |
| *B2m* | CTTTCTGGTGCTTGTCTCACTGA | GTATGTTCGGCTTCCCATTCTC |
| *Ppia* | TTCCTCCTTTCACAGAATTATTCCA | CCGCCAGTGCCATTATGG |
| *Hprt* | TGGATATGCCCTTGACTATAATGAGTAC | AGGACTCCTCGTATTTGCAGATTC |

**Table S2: List of primary antibodies.**

| **Primary Antibodies** | **Supplier** | **Cat. No.** |
| --- | --- | --- |
| ULK | Cell Signaling | 8054 |
| p-ULK (ser757) | Cell Signaling | 6888 |
| FoxO1 | Cell Signaling | 2880 |
| p-FoxO1 (Ser256) | Cell Signaling | 9461 |
| Akt | Cell Signaling | 9272 |
| p-Akt (Ser473) | Cell Signaling | 9271 |
| S6 | Cell Signaling | 2317 |
| p-S6 (Ser235/236) | Cell Signaling | 4856 |
| 4E-BP1 | Cell Signaling | 9452 |
